# Sex-specific expression of alternative transcripts in *Drosophila*

**DOI:** 10.1186/gb-2006-7-8-r79

**Published:** 2006-08-25

**Authors:** Lauren M McIntyre, Lisa M Bono, Anne Genissel, Rick Westerman, Damion Junk, Marina Telonis-Scott, Larry Harshman, Marta L Wayne, Artyom Kopp, Sergey V Nuzhdin

**Affiliations:** 1 Department of Molecular Genetics and Microbiology, 1376 Mowry Road room 116, University of Florida, Gainesville, FL 32611, USA; 2 Computational Genomics, 901 West State Street, Purdue University, West Lafayette, IN 47907, USA; 3 Section of Evolution and Ecology, One Shields Avenue, University of California, Davis, California 95616, USA; 4 Department of Horticulture, 625 Agriculture Mall Dr., Purdue University, West Lafayette, IN 47907, USA; 5 Department of Agronomy, 915 West State Street, Purdue University, West Lafayette, IN 47907, USA; 6 Department of Zoology, 223 Bartram Hall, University of Florida, Gainesville, FL 32611, USA; 7 School of Biological Sciences, 335 Mant, University of Nebraska, Lincoln, NE 68588, USA; 8 Center for Genetics and Development, One Shields Avenue, University of California, Davis, California, 95616, USA

## Abstract

**Background:**

Many genes produce multiple transcripts due to alternative splicing or utilization of alternative transcription initiation/termination sites. This 'transcriptome expansion' is thought to increase phenotypic complexity by allowing a single locus to produce several functionally distinct proteins. However, sex, genetic and developmental variation in the representation of alternative transcripts has never been examined systematically. Here, we describe a genome-wide analysis of sex-specific expression of alternative transcripts in *Drosophila melanogaster*.

**Results:**

We compared transcript profiles in males and females from eight *Drosophila *lines (*OregonR *and *2b*, and 6 RIL) using a newly designed 60-mer oligonucleotide microarray that allows us to distinguish a large proportion of alternative transcripts. The new microarray incorporates 7,207 oligonucleotides, satisfying stringent binding and specificity criteria that target both the common and the unique regions of 2,768 multi-transcript genes, as well as 12,912 oligonucleotides that target genes with a single known transcript. We estimate that up to 22% of genes that produce multiple transcripts show a sex-specific bias in the representation of alternative transcripts. Sexual dimorphism in overall transcript abundance was evident for 53% of genes. The *X *chromosome contains a significantly higher proportion of genes with female-biased transcription than the autosomes. However, genes on the *X *chromosome are no more likely to have a sexual bias in alternative transcript representation than autosomal genes.

**Conclusion:**

Widespread sex-specific expression of alternative transcripts in *Drosophila *suggests that a new level of sexual dimorphism at the molecular level exists.

## Abstract 2

A genome-wide microarray analysis of sex-specific expression of alternative transcripts in Drosophila shows sexual dimorphism in transcript abundance for 53% of the genes.

## Background

Microarray hybridization, with its unprecedented ability to monitor genome-wide gene expression profiles, is paving the way for exploring previously intractable problems in developmental biology [1-5], neurobiology and behavior [6-8], evolutionary genetics [9-13], and other areas of biology. One of the technology's most exciting applications lies in establishing an experimental and theoretical framework for linking genetic variation in transcript abundance and phenotypic traits [14-19]. However, there is more to the regulation of gene expression than steady-state transcript abundance. In particular, many multi-exon genes in eukaryotic genomes are subject to alternative splicing, which is thought to increase phenotypic complexity by producing multiple, functionally distinct proteins [20-24]. Much of this alternative splicing may be tissue-specific, introducing an additional layer of regulatory complexity [22,25]. Sexual dimorphism and genetic variation in alternative splicing have never been systematically examined, but it is reasonable to expect that such variation would have a considerable impact on phenotypic diversity.

To estimate the extent of sexual dimorphism and genetic variation in the production of alternative transcripts, we designed a new *Drosophila *whole-genome microarray that allows us to distinguish multiple transcripts of many genes using long (60-mer) oligonucleotide probes. Since genome annotation changes frequently as more data become available, we have created a flexible, easily updated design, and developed software that allows automatic annotation updates. We have used the new platform to compare gene expression profiles of males and females in eight lines of *Drosophila melanogaster*, and found that over 50% of all genes are expressed in a sex-biased manner. Interestingly, we estimate that between 11% and 24% of *Drosophila *genes known to produce multiple transcripts show sexual bias in the expression of alternative transcripts.

## Results

RNA was extracted from male and female flies from two laboratory lines of *D. melanogaster*, *OregonR *and *2b*, and six randomly chosen recombinant inbred (RI) lines derived from these parents. We detected 8,292 genes with a single known transcript, represented by 8,310 microarray probes, in at least one line/sex combination. In addition, an additional 1,651 multi-transcript genes and 71 gene families were each represented by a single hybridizing probe, since some of the probes targeting alternative transcripts and gene families were not detected in this experiment. These 10,014 transcripts were analyzed using the ANOVA model for single transcripts (see Materials and methods). Of these transcripts, 56% showed significant variation at a false discovery rate (FDR) of 0.05 (Table 1), with the vast majority of this variation attributable to differences between males and females (5,221 out of 10,014 transcripts). Among these sex-biased genes, 56% were expressed at a higher level in females than in males. Among lines, 349 transcripts showed significant differences (Table 1), and only 1 (*CG33092*) showed a significant difference in the interaction between line and sex.

For 828 of the 2,479 genes known to produce multiple transcripts, microarray probes targeting 2 or more distinct sets of transcripts showed detectable hybridization. These probes were analyzed using the ANOVA model for multiple transcripts. Expression levels of 653 (78%) of these genes showed significant variation at the FDR of 0.05, with the majority (544) showing a sex bias and 202 showing significant differences among lines (that is, genetic variation). For 91 gene families, hybridization was detected for probes targeting two or more sets of transcripts. Of these, 79 were variable, with 67 of these showing significant differences between males and females. For one transcript (*modulo*), the direction of the difference between males and females was affected by genotype.

### Validation of platform

To evaluate the performance of the new microarray platform, we analyzed the expression of genes for which we had *a priori *expectations of sex-biased expression. First, we examined components of the somatic sex determination pathway and its known downstream targets [26,27]. As expected, the female-specific genes *transformer *and *yolk proteins 1*, *2*, and *3*, each represented by a single probe on our arrays, showed significantly female-biased expression in our experiments (Table 2). Female-biased expression was also observed for *hermaphrodite *and *transformer 2 *(*tra2*), which are expressed in both sexes. *tra2 *was represented by four hybridizing probes that targeted different regions of a nearly identical set of transcripts; all of these probes showed similar ratios of expression in males and females (Table 2). *doublesex *(*dsx*) is spliced in a sexually dimorphic manner, producing a male-specific and a female-specific transcript [28]. In our design, *dsx *was represented by four probes: one targeting a male-specific exon, one targeting a female-specific exon, and two targeting an exon common to male and female transcripts. We found that the male-specific probe indeed showed male-biased expression, the female-specific probe showed female-biased expression, and the common probes showed expression levels intermediate between the two sex-specific probes (Table 2). These results indicate that, as intended, the new microarray platform can distinguish among different exons and thereby reliably indicate alternative transcript production.

Next, we retrieved from FlyBase a list of genes known to be involved in the development or function of reproductive organs. We subdivided this list into three non-overlapping sets: genes known to function only in the female reproductive system (565 microarray probes, representing 326 genes), those known to function only in the male reproductive system (60 probes/42 genes), and genes implicated in both male and female reproductive systems (120 probes/86 genes). Most of these genes, however, are not exclusive to the reproductive system and are expressed in a wide range of non-reproductive organs as well. Since our experiments utilized whole-body RNA samples, we may not always be able to detect sex-biased expression in the reproductive organs. We found that among the female reproductive system genes, 86% were female-biased, with 72.5% being significant for sex and/or sex-by-probe interaction effect (Additional file 1). Conversely, among the male reproductive system genes, 64.3% were male-biased, with 55.5% showing significant sex effect (Additional file 1). We also analyzed a set of genes that are thought to be expressed only in males. These genes included a number of secreted accessory gland proteins [29-31], putative odorant-binding proteins expressed in male-specific chemosensory organs [25], and sperm-specific structural proteins [32]. We found that 100% of these genes (11 out of 11) showed male-biased expression in our experiments (Additional file 1). Finally, we examined a set of male-specific transcripts identified earlier by differential cDNA hybridization [33,34], and found that all genes detected in our experiments (ten out of ten) showed male-biased expression (Additional file 1). Finally, we examined the expression of six *Y*-linked genes represented on our arrays. Only two of them were expressed at detectable levels in enough samples to be considered informative. As expected, neither was present in any female samples, but both were detected in the majority of male samples. Together, these analyses confirm that the new microarray platform is effective for detecting sex-biased gene expression. For genes that produce multiple transcripts due to alternative splicing, or due to the presence of multiple transcription initiation or termination sites, we tested whether the relative proportions of alternative transcripts differed between sexes or lines. We used the ANOVA model for multiple transcripts (see Materials and methods) to examine the genes for which at least two probes targeting distinct sets of transcripts produced detectable hybridization. For these genes, we tested whether the relative amounts of signal from the different probes differed between sexes or lines. Such differences (called sex-by-probe or line-by-probe interactions) imply that the same gene produces alternative transcripts in different amounts in males versus females, or in different genotypes, respectively.

Sex-specific production of alternative transcripts has previously been reported for only a handful of genes, so we lack an extensive set of positive controls against which to compare our results. The best-known example in *Drosophila *is the *dsx *gene [28]. Indeed, as shown above, probes targeting the male- and female-specific exons of *dsx *show different expression levels in different sexes (Table 2). When analyzed using the ANOVA model for multiple transcripts, the *dsx *gene shows a significant sex-by-probe interaction (*P *< 0.0001; Table 2). *Sex-lethal *(*Sxl*), which also produces male- and female-specific alternative transcripts [35], was represented in our experiments by five probes targeting different subsets of transcripts, and also showed significant sex-by-probe interaction (Table 2). These results suggest that our platform has the power to detect quantitative differences in the relative amount of alternative transcripts in different sexes.

### Sex-specific expression of alternative transcripts

We examined 828 genes for which 2 or more probes representing distinct sets of transcripts showed detectable hybridization. Of these, 182 (22%) showed significant sex-by-probe or line-by-probe interactions at the FDR of 0.05, indicating that the relative amounts of alternative transcripts were different in males and females, or in different lines (Table 3). For the vast majority of these genes (177 out of 182 genes), the differences were attributable to sex. These genes had a variety of molecular functions, including transcription factors, cell signaling components, cytoskeletal proteins, and others (Additional data files 2 to 4). Of the 828 multi-transcript genes, 55 had 2 or more probes targeting different subsets of transcripts, but no probes targeting the entire set of transcripts produced by the locus (that is, 'local' probes only; see Materials and methods). Among such genes, 19 (35%) showed evidence of sex-specific or line-specific bias in the production of alternative transcripts (Table 3). Interestingly, no obvious relationship was observed between the number of probes targeting a given gene and the likelihood of finding evidence for sex-specific transcript representation.

**Figure 1:**
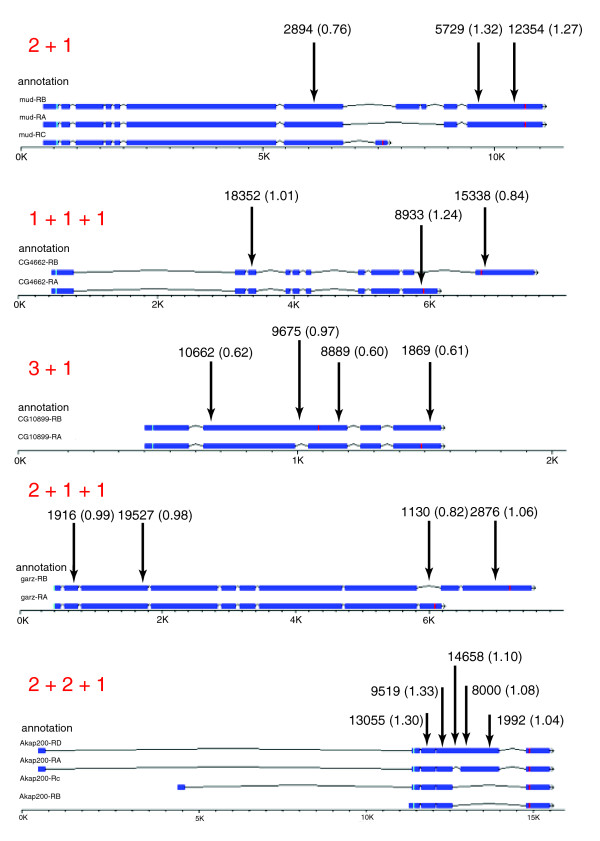
Experimental approach used to detect sex-specific splicing. Probes designed based on sequence clustering may target either constitutive or alternatively transcribed exons. Each panel shows a different example of probe distribution among constitutive and alternatively transcribed regions. For instance, '2+1+1' indicates that the corresponding gene has two probes targeting a common region and one probe targeting each of two alternatively transcribed regions, '3+1' indicates that the gene has three common probes and one probe that targets an alternatively transcribed region, and so on. For each probe, the figure shows its designating number, location in the transcript, and the ratio of the normalized and log-transformed (natural log) values between females (numerator) and males (denominator). Note that different probes that target the same subset of transcripts have similar values for the normalized log transformed male/female expression ratios, even if they are located in different exons. In contrast, probes that target alternatively spliced regions have different values for the normalized log transformed male/female expression ratios.

To examine sex-specific expression of alternative transcripts more closely, we analyzed the set of 177 genes that showed significant sex-by-probe interactions on a probe-by-probe basis (Additional file 5). In general, we found that probes targeting the same exon, or different constitutively spliced exons, tended to have similar male/female expression ratios (Figure 1). Conversely, probes targeting different exons tended to have expression ratios that were different from each other and from constitutively spliced exons (Figure 1).

We did observe some exceptions where different probes targeting the same set of annotated transcripts showed different male/female expression ratios (Additional file 5). Such exceptions could be due either to intrinsic biases in probe hybridization, or to mistakes in the current FlyBase annotation (that is, exons indicated as constitutive might in fact be subject to alternative splicing or transcription). To estimate the extent to which our results may be affected by these factors, we used the ANOVA model for multiple transcripts to compare probes that, according to the current annotation, targeted different regions of the same set of transcripts. This control allows us to assess the maximum proportion of significant sex-by-probe or line-by-probe interactions expected in the absence of differential transcript production (see Materials and methods). Of the 1,321 control probe sets, 129 (9.77%) showed significant interactions - a proportion that is well short of the 22% found for probes targeting distinct sets of transcripts. This suggests that although intrinsic probe biases and/or mistakes in the annotation may have an effect, this effect is not sufficient to explain the observed variation in relative transcript abundance. We conclude that a large proportion of multi-transcript genes in the *Drosophila *genome produce alternative transcripts in a sexually dimorphic manner.

### Confirmation of sex-specific alternative splicing by quantitative PCR

**Figure 2:**
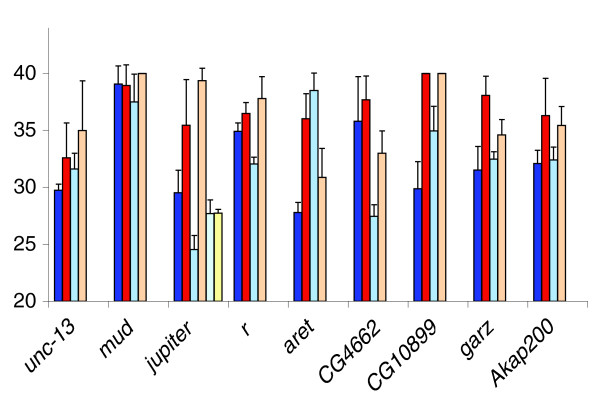
Sex-specific amplification of alternative transcripts from nine genes that showed significant sex by probe interaction in the microarray data (*unc-13*, *mud*, *jupiter*, *r*, *aret*, *CG4662*, *CG10899*, *garz*, *Akap200*; see Table 3). The graph shows the average CTs for each exon junction in males and females of the *OregonR *line. CT values were calculated by performing qPCR with SYBR^® ^Green I dye chemistry on three bioreplicates consisting of four virgin males and females, and correspond to the number of cycles when the fluorescence intensity was significantly above background during the exponential phase of amplification; dark blue, male transcript 1; light blue, male transcript 2; green, male transcript 3; red, female transcript 1; pink, female transcript 2; orange, female transcript 3.

Several genes that showed significant sex-by-probe interactions were further tested using quantitative rt-PCR (qPCR) with primers that flanked exon junctions. First we evaluated the ability of qPCR to detect sex-biased transcript abundance. The genes *CG7441*, *Sxl*, *fru*, and *Nep4*, which showed evidence of sex-specific expression in the microarray data, were used as positive controls, while *Lsp1beta*, which was not sex-biased on the array, was used as a negative control. In all cases, qPCR results were consistent with array results (Additional file 6). We then designed two to three primer pairs for each of nine genes that are known to be alternatively spliced and that showed evidence of sex-specific splicing in microarray experiments: *unc-13*, *mud*, *Jupiter*, *r*, *aret*, *CG4662*, *CG10899*, *garz*, and *Akap200*. These primer pairs were designed to amplify either constitutive exon junctions, or alternative splice junctions that were present in non-overlapping sets of transcripts. We measured the cycle thresholds of amplification (CT) for each primer pair in males and females of the *Oregon-R *line, and tested whether these values showed significant sex-transcript interaction. Such interaction would indicate that different exons were produced in different amounts in males versus females, confirming the microarray results. We observed statistically significant differences in transcript ratios in males versus females for eight out of nine genes (Additional data file 6; Figure 2). For the ninth gene, *Akap200*, transcript ratios also differed in the predicted directions, but the ANOVA interaction term was not statistically significant.

### Genomic distribution of differentially expressed genes

We tested whether the genes that showed evidence of differences in gene expression were more likely to be located on the *X *chromosome than on the autosomes using a χ^2 ^test. For single-transcript genes, 57% (840) of the *X*-linked genes showed a significant difference in gene expression among sexes or lines, compared to 54% (4,630) for the autosomal genes. This difference, while slight, is greater than expected by chance (*P *= 0.0260). In other words, *X*-linked genes are significantly more likely to show differences in gene expression than autosomal genes. We then tested whether male- and female-biased genes were distributed in the same proportions between the *X *chromosome and the autosomes. We identified 559 female-biased genes on the *X *chromosome and 2,466 on the autosomes, compared to 281 *X*-linked and 2,164 autosomal male-biased genes. Thus, 18.5% of all female-biased genes are located on the *X *chromosome, while for male-biased genes the corresponding number is only 11.5%. This difference is highly significant (*P *< 0.0001), demonstrating that the X chromosome is enriched for female-biased single transcript genes.

The same comparisons were performed for multi-transcript genes. There were 116 *X*-chromosomal and 616 autosomal genes that showed a significant difference in gene expression in either sex or line; these showed no statistical evidence for chromosomal bias (*P *= 0.9479). However, among genes that showed sex-biased transcript abundance, 78 *X*-linked and 304 autosomal genes were female-biased, compared to 38 *X*-linked and 312 autosomal genes that were male-biased. The proportions of female- and male-biased genes located on the *X *chromosome (20.4% and 10.9%, respectively) were significantly different (*P *= 0.0004), demonstrating that the X chromosome is enriched for female-biased multi-transcript genes.

We also tested whether sex-specific production of alternative transcripts (significant sex-by-probe interaction in the ANOVA model for multiple transcripts) was more likely to be observed for *X*-linked than for autosomal genes. There were 28 *X*-linked and 177 autosomal genes that showed significant sex-specific transcription; this proportion was not significantly different from that expected given the relative abundance of genes on the *X *chromosome and the autosomes (*P *= 0.3221). The male/female bias in alternative transcript representation was also independent of chromosomal location (*P *= 0.3479).

## Discussion

### The benefits of microarray design based upon sequence similarity

To perform a quantitative analysis of alternative transcript expression, we have designed transcript-specific probes based solely on sequence clustering (see Materials and methods). Definitions based on biological constructs such as exon junctions impose design restrictions that may result in probes that cross-hybridize to multiple genes, or do not have optimal hybridization properties with their intended targets. In contrast, our approach allows us to select probe sequences that will hybridize only to single transcripts. Our analysis shows that such probes perform in a uniform and highly reproducible fashion (Table 4). Moreover, a design based on the exon/intron structure of genes would require frequent revision to reflect changes in the genome annotation, whereas definitions based on sequence similarity are likely to change less frequently. A limitation to this design is that a gene nested in the intron of another gene can be difficult to distinguish from an alternative exon in the absence of junction information. We have based our microarray design on FlyBase v3.1 annotation [36]. To keep pace with annotation updates, we have developed software that tracks the latest FlyBase annotation of the probes comprising our microarrays (or any other oligonucleotides). This insures that, as the understanding of the genome evolves, the classification of probes can be updated as well. The result is a flexible platform that will enable researchers to perform simultaneous analysis of transcription and alternative transcript production on a genome-wide basis.

### Sex-specific gene expression

A very large fraction of the genome appears to be differentially expressed between males and females. In our experiments, 53% of all expressed genes (5,832 out of 10,933, including 291 unannotated genes) showed sex-biased expression. Other studies utilizing different microarray platforms produced very similar estimates [19,37-42]. It is worth observing that all these studies, like ours, were performed in sexually mature, intact adults, and it is not surprising that gene expression profiles at this stage are dominated by the reproductive differences between males and females. It is clear, however, that most of the sexual dimorphism in gene expression is due to the germline. Comparisons of gonadectomized adults, or adults in which germ cells have been genetically ablated, produce much lower estimates of sexual dimorphism, on the order of 1.5% to 3% [1,41]. Sexually dimorphic gene expression is much more prevalent in the germline than in the soma not only in *Drosophila*, but also in *Caenorhabditis elegans *[43-45] and in the mouse [46]. This pattern is observed despite the differences in the mechanisms of sex determination in these taxa: in flies, the sex of each individual somatic cell is determined autonomously [47], whereas in mammals somatic sexual differentiation is controlled by a global hormonal mechanism [48].

We find that more genes show female-biased than male-biased expression (55% versus 45%). This result is in agreement with some of the previous reports [39], although other studies suggest that male-biased expression is more common than female-biased expression [41]. The reasons for this contradiction are not clear, and could in principle include different lines, different microarray platforms, and/or different statistical approaches. However, many of the genes that showed significant differences in expression between males and females in our experiments were also found to be sexually dimorphic in other studies [19,37-40]. Interestingly, we found that female-biased genes were much more likely to be located on the *X *chromosome than male-biased genes (18.5% versus 11.5% for single-transcript genes and 20.4% versus 10.9% for alternatively spliced genes; *P *< 0.0001). Similar 'feminization' of the *X *chromosome has previously been observed in *Drosophila *[40,41] and *C. elegans *[44,45].

We found that only two genes, *modulo *and *CG33092*, show significant sex differences that change depending on the line examined (that is, have genetic variation for sex dimorphism). In contrast, some earlier reports suggested that as much as 10% of the genome may show such sex-genotype interactions [37,38]. This is despite the fact that the lines used in this study included the two parental lines used in one of these studies [38], as well as recombinant inbred lines derived from these two parents. The most likely reason for this is that significance thresholds used in our analysis were much more stringent than in previous reports. In fact, if we use the nominal significance threshold of 0.01, as in those reports, we find approximately the same proportions of genes showing sex-by-line interactions (not shown). We have chosen to report FDR-corrected thresholds since this approach incorporates an appropriate correction for multiple testing. It is also important to note that this study examines a limited number of lines, the two parents *OregonR *and *2b *and six recombinant offspring from these two parents. The extent of alternative transcript production among lines will only be clear as more lines are sampled.

### Evidence for functional consequences of alternative splicing

A large proportion of multi-exon genes in animal genomes are alternatively spliced, with estimates ranging from 30% to over 90% [20-24]. Alternative splicing is thought to make a significant contribution to phenotypic complexity by allowing a single locus to produce multiple, and possibly functionally distinct, proteins [49-52]. Supporting this view, many of the alternatively spliced genes in the human genome are spliced in a tissue-specific manner [25]. In *Drosophila*, alternative splicing plays a prominent role in development, most notably by controlling sex determination [53-55]. In at least some *Drosophila *genes, alternative splicing is regulated in a sex-, tissue-, and/or stage-specific manner, so that different subsets of proteins encoded by the locus are produced in different developmental contexts [53,56-61]. Alternatively spliced protein isoforms can, at least sometimes, have distinct functional specificities. For example, alternative isoforms of the *lola *transcription factor have different functional domains, and mutations affecting the different isoforms have distinct phenotypes [57]. Similarly, one of the alternatively spliced transcripts of the *Drosophila *tyrosine hydroxylase (*pale*) is required for cuticle development, while a different transcript functions primarily in neurotransmission [62]. One dramatic example of alternative splicing is the cell adhesion receptor *Dscam*, which may produce up to 38,016 splicing variants [63,64]. Recent evidence indicates that specific isoforms function in distinct axon guidance pathways [65]. However, evidence of the functional impact of alternative splicing remains largely anecdotal, and for the vast majority of genes functional comparisons between alternatively spliced variants are yet to be performed. At present, the extent to which alternative splicing contributes to functional protein diversity remains a matter of speculation. Exon-specific RNA interference [66] may finally allow this question to be addressed in a systematic manner.

We used the new microarray platform to estimate the extent of sex-specific production of alternative transcripts in the *Drosophila *genome. Approximately 22% of multi-transcript genes showed significant evidence that alternative transcripts were present in different ratios in males versus females. Some of these results might be experimental artifacts due to technical differences between probes, or mistakes in the current gene annotation. To address this concern, we used identified multiple probes that were predicted to hybridize to the same target transcripts as controls. Significant interactions between sex and probe will provide an estimate of the maximum proportion of significant tests that might be due to differences among probes, or problems with annotation. We found this proportion to be less than 10%, suggesting that at least 12% of all genes that produce alternative transcripts do so in a sex-specific manner. qPCR with primer pairs flanking alternative exon junctions confirmed sex-biased splicing for eight out of nine tested genes, indicating that exon-specific microarray probes provide a reliable means of detecting variation in the relative abundance of alternative transcripts. As in the case of sex-biased transcription, we suspect that much of the sex-specific splicing may be accounted for by reproductive tissues, and that most differences between males and females are likely to be quantitative rather than qualitative. Despite these qualifications, the prevalence of sexual differences in the production of alternative transcripts may have important functional consequences, and needs to be investigated in greater detail.

The *Drosophila *genome contains a number of RNA-binding proteins that function as splicing regulators *in vivo *[67]. Importantly, some of these proteins appear to be required for alternative splicing. In particular, several of them are essential components of protein complexes that carry out sex-specific splicing of *dsx *and *Sxl *[68-71], while RNAi-induced knock-down of the *pasilla *and *mub *genes disrupts the splicing of specific exons in the *para *and *Dscam *transcripts [67]. Thus, it is easy to envision a mechanism for sex-, tissue-, and stage-specific regulation of alternative splicing through differential expression of RNA-binding proteins. Indeed, we found that 95% (35 out of 37) of splicing regulators previously characterized [67] are expressed at significantly different levels in males and females at a FDR of 0.05 (Table 5). This proportion is much greater than the overall frequency of sex-biased gene expression in the *Drosophila *genome (approximately 53% in this study). We hypothesize that sex-specific expression of splicing regulators contributes to the prevalence of sex-specific production of alternative transcripts observed in our experiments. One attractive use of the new microarray platform would be to jointly monitor the expression of splicing regulators and the alternative transcripts of their target loci in different developmental contexts (tissues, sexes, and stages) and in different lines.

## Materials and methods

### Transcript clustering and probe design

**Figure 3:**
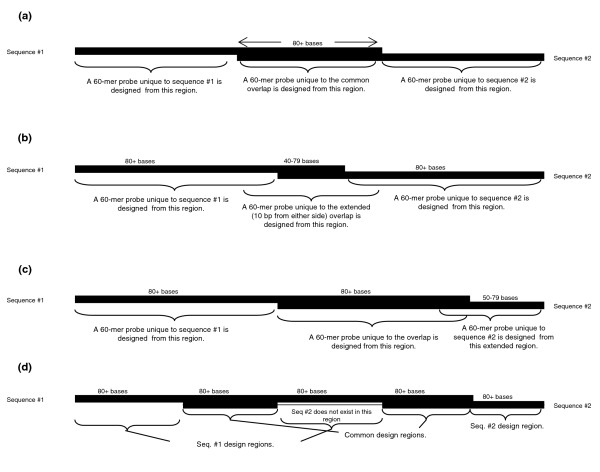
Examples of transcript clustering. Transcripts were clustered by BLAT and then aligned in ClustalW. Some of the more common clustering patterns are depicted. **(a) **Two transcripts, each with a unique region of at least 80 bases and a common region of at least 80 bases; **(b) **two transcripts, each with a unique region of at least 80 bases, and a common region between 40 and 79 bases; **(c) **two transcripts with a common region of at least 80 bases, a unique region of at least 80 bases and a unique region of at least 50 bases; **(d) **two sequences with a gapped alignment.

Our goal was to design microarray probes capable of distinguishing alternative transcripts, as well as members of multi-gene families. In order to maximize probe specificity, we first examined sequence similarity among all known and predicted transcripts of *D. melanogaster*. Sequences of 18,187 transcripts, including 16,064 transcripts annotated in FlyBase [36] and 2,123 predicted transcripts [72], were obtained in the fall of 2004, and 440 additional transcripts in the Spring of 2005 (FlyBase version 3.1). Gene and transcript identity was tracked through all following analyses using their CG numbers - unique identifiers assigned by the FlyBase [36]. We identified and removed 160 duplicate transcripts. The remaining 18,027 transcripts were compared among themselves using BLAT v29 [73] to identify regions of sequence similarity. This clustering resulted in a division of the transcriptome into two groups - 'singletons' and 'clusters'. The former group consisted of 13,069 transcripts that did not show sequence similarity to any other transcript, while the latter consisted of 4,958 transcripts that showed sequence similarity to at least one other transcript. We deliberately did not exclude paralogous genes from this clustering, as we wished to design probes targeting the most diverged regions of such genes. Each transcript cluster was aligned using ClustalW v1.8 [74]. Sequences that were shared by two or more transcripts were designated as 'common' regions, while regions that showed no similarity to other transcripts were designated as 'unique'. There were many possible scenarios for the alignment of transcripts within a cluster, some of which are illustrated in Figure 3. Some clusters displayed more complex relationships, including cases where the transcripts had no single region that was common to all of them, but did have several regions that were each shared by a different subset of transcripts. In these and other difficult cases, sequence alignments were performed manually. No *a priori *information about the exon/intron structure of the genes was used during cluster alignment. The overall set of 4,958 clustered transcripts contained 2,720 common and 2,545 unique regions. For most transcript clusters, common and unique regions identified by sequence alignment correspond to constitutively and alternatively spliced exons, respectively. Some examples of this correspondence are shown in Figure 1.

For each singleton transcript, and each unique and common region of clustered transcripts, we designed at least one 60-mer oligonucleotide probe. For 1,929 common regions of sufficient length to support non-overlapping probes that fit our design criteria, we designed two probes per region. To select the probes, we examined all possible 60-mers for each of the target regions, and scored each candidate based on several criteria, including GC content, OligoArray 2.1 score [75], homopolymer length, dimer formation, and self-complementarity. Probes that satisfied all optimality criteria could be designed for all but 312 target regions. For those regions, multiple non-optimal probes were selected. All probes were examined to verify that they matched only the expected regions in the current version of *Drosophila *genome annotation, and subjected to a final BLAT verification. In particular, probes that were designed for singletons or unique regions were checked to make sure they did not match any other transcripts, whereas probes that were designed to represent common regions were confirmed to match only the expected set of transcripts.

The resulting microarray design included 12,994 probes that targeted singleton transcripts (Table 6). If the current FlyBase annotation is correct, these transcripts represent genes that are not subject to alternative splicing. Most of these transcripts (12,912) were each represented by a single probe, while 37 were represented by multiple probes (for a total of 82 probes). Clustered transcripts were subdivided into two further categories. The smaller category consisted of 177 clusters where at least one probe matched more than one CG number in the latest FlyBase annotation. Each of these clusters was assumed to represent a paralogous 'gene family', and probes targeting common and unique sequences were designed for each such cluster for a total of 566 probes. In the larger category, 2,768 clusters represented by 7,207 probes each consisted of multiple transcripts designated by the same CG number in FlyBase, and thus corresponded to the same gene (Table 6). We refer to such genes as 'alternative transcripts', as in some cases the production of multiple transcripts is due not to differential splicing, but rather to utilization of different transcription initiation or termination sites. The alternative transcripts were targeted by probes belonging to two distinct types. Again, 'common' probes represent sequences found in more than one transcript, while 'unique' probes represent sequences found in only one transcript. Probes common to all transcripts in a cluster were designated as 'global', while those representing only a subset of transcripts, or a single transcript, were designated as 'local'.

We used the human genome to design 20 negative control probes according to the same criteria as the *Drosophila *probes. These probes were compared to the *Drosophila *genome sequence to verify that they had no sequence similarity to any *D. melanogaster *genes. Five of these negative controls were randomly chosen for printing, and each was placed on the microarray one hundred times. At the end of the design there were an additional 3 spots available upon which negative controls were placed, for a total of 503 negative control spots. These negative controls allow us to estimate the distribution of signal intensities for probes that fail to hybridize, and to make present/absent calls for each transcript.

The microarray printed according to our design by Agilent Technologies had a total of 22,575 spots, including 20,768 spots representing *Drosophila *transcripts, 503 negative control spots, and 1,304 Agilent controls (Table 6). These chips can be ordered from Agilent directly by quoting the AMADID number 012798.

### Annotation and update procedure

Genome annotation changes as gene prediction methods improve and more experimental data become available. To allow the microarrays to be regularly updated to reflect these changes, we have written an automated annotation program that tracks the identity of each probe in the current version of FlyBase, and reports how many transcripts match this probe and whether this set is concordant with the expected design. We output all matches between probes and transcripts and then reduce this information to one row per probe, with a column that lists all matches for that probe. Detailed annotation is extracted for the first match, using CG numbers to identify which gene(s) are targeted by each probe. Other columns enumerate the number of transcripts predicted for that CG in the current annotation, the number of transcripts the particular probe matches, the number of probes for that CG in the current microarray design, and whether the probe aligns with the gene with which it was originally designed to align. In this last column, four different designations may be given: 'match' (probe aligns with the same CG as expected), 'mismatch' (a different CG than expected), 'extended' (same CG as expected, but the probe hits more transcripts of that CG than expected), and 'not found' (no matches to any transcripts in the current FlyBase). Since the initial design includes predicted but unconfirmed genes, we expect that some probes will not be found in the current database. Additionally, probes are categorized into one of the following groups: 'singletons' (one match per probe), 'gene families' (match to more than one CG number), 'alternative transcripts' (one CG number represented by multiple common and unique regions), and 'pseudo-clusters' (more than one probe representing a single transcript). If two or more probes in an alternative transcript or gene family hit the same target region in the current annotation file, these probes were considered part of a 'set'. Each such set can then be classified as 'global' (expected hybridization to all transcripts of a particular transcript identified by a CG designation), or 'local' (expected hybridization to a subset of alternative transcripts of a specific CG designation).

### *Drosophila *lines and RNA sample preparation

Experiments were conducted on flies from two standard laboratory strains of *D. melanogaster*: *OregonR *[76] and *2b *[77], and six randomly chosen recombinant inbred (RI) lines derived from these parental lines [78]. Each of the 8 lines was grown in 4 separate replicates of small mass-matings containing, on average, 20 adults, with a sex ratio of 1:1. Bottles were maintained at 25°C with a 12:12 hour light:dark cycle, and the parents were removed after 3 days. We collected 20 virgin males and females within 24 hours from each replicate, transferred separately to fresh vials, and maintained for 3 days. After the maturation period, the virgin adult females and males were snap-frozen in liquid nitrogen for total RNA extraction.

RNA was extracted from each sample using Trizol reagent (Invitrogen Carlsbad, California, USA) according to the manufacturer's instructions, and purified using RNAeasy Kit (Qiagen, Valencia, CA, USA). RNA concentration was determined using NanoDrop Spectrophotometer (NanoDrop Technologies, Inc. Wilmington, DE USA), and the sample quality was examined using the Agilent 2100 Bioanalyzer (Agilent Technologies, Inc. Palo Alto, CA USA). We used 500 ng of RNA from each sample for the microarray experiment.

### Microarray hybridization and signal detection

Fluorescent cRNA was synthesized using the Aglient low RNA input fluorescent linear amplification kit following the manufacturer's protocol. Briefly, first and second strand cDNA was synthesized from 500 ng total RNA using an oligo dT-promoter primer and reverse transcriptase. Next, cRNA was synthesized using a T7 RNA polymerase, which simultaneously incorporates cyanine 3- or cyanine 5-labeled CTP. Labeled RNA was cleaned using Qiagen RNeasy columns and cRNA yield was quantified on a NanoDrop ND-1000 spectrophotometer. We pooled 750 ng of each labeled sample and hybridized to the arrays following the manufacturer's protocol. Hybridizations were performed with males and females of the same line labeled in contrasting dyes and hybridized to the same chip. We analyzed four independent biological replicates for each line and sex combination. For two of these replicates, males were labeled with Cy3 and females with Cy5, whereas for the other two the dyes were reversed. No technical replicates were performed as reliability of the Agilent platform is, on average, above 90% (unpublished data by LMM, MLW, SVN, LH, AK). This design maximizes the ability to test for sex effects (NIH project 5R24GM065513), and ensures that effects of sex remain balanced in the event of chip failure.

Microarray experiments were carried out at the Interdisciplinary Center for Biotechnology Research Microarray Core, University of Florida. Hybridization occurred for 17 hours at 60°C in accordance with the manufacturer's instructions, and arrays were scanned using an Agilent Microarray scanner. There were seven technical failures, which were unrelated to the platform, leaving 25 successful hybridizations. Additionally, Agilent reported a manufacturing error that affected 2,310 spots on each chip, including 150 of the 503 negative controls. The failed chips and defective spots were removed from further consideration.

Images were analyzed using Imagene software version 6.0 at the Purdue University Genomics Database Facility. Spots were individually quantified, and the mean intensities and mean background signal corresponding to each spot were exported into .csv files. As with other chip analysis software, in Imagene, the units are a function of pixel intensity. Individual files were collated for analysis at the Purdue University Genomics Database Facility. Transcript abundance was estimated as the natural log of the spot mean minus the mean of the local background.

All spots on the array were compared between pairs of biological replicates to determine the reproducibility of RNA labeling and hybridization. Weighted kappa values ranged from 0.754 to 0.906, with a median of 0.85 (Table 4), indicating that our platform had high repeatability; in general, weighted kappa values above 0.75 are considered excellent [79]. Following this overall assessment, we examined repeatability for subsets of probes to determine whether any of the known variables (including GC content, Tm, Oligoarray score, the number of probes per CG, the number of transcripts per probe, and whether the probe hybridized to multiple CGs) affected the reproducibility of hybridization. For most comparisons, these variables had little to no impact on the concordance among replicates. Additionally, the few probes that were designed outside of the usual stringent criteria did not perform worse than the optimally designed probes (median weighted kappa of 0.83). However, there were three large clusters of alternative transcripts (consisting of 11, 16, and 26 transcripts) that produced inconsistent results across replicates.

We then examined the distribution of signal intensities for the 353 negative control spots. These spots form the null distribution of intensity values for a given slide and dye combination in the absence of hybridization. Individual *Drosophila *probes were declared to have hybridized if the intensity of that spot was greater than the intensity of 95% of the negative controls for that slide and dye combination. Probes were considered to be detected for a particular treatment (that is, line/sex combination) if they hybridized in 50% or more of the replicates of that treatment. Probes that were not detected in at least one treatment were considered uninformative, and not considered further. The 20,265 available spots represented three groups of probes: Agilent controls (1,172 spots), negative hybridization controls (353 spots), and *Drosophila *probes (18,740 spots). There were 13,874 *Drosophila *probes (74%) found to hybridize in at least one treatment, including 187 of the 311 suboptimal probes (Table 6). Of the 2,156 probes designed for predicted genes, 963 showed detectable hybridization, confirming the existence of predicted transcripts. Of the 13,874 probes that were detected in at least one treatment, 5,128 represented alternative transcripts (2,479 genes), 436 represented gene families (162 genes), 45 represented pseudo-clusters (27 genes), and 8,265 represented singleton transcripts (8,265 genes). The data discussed in this publication have been deposited in NCBIs Gene Expression Omnibus and are accessible through GEO Series accession number GSE4976.

### Statistical analyses

For genes that had more than one informative transcript, the following ANOVA model for multiple transcripts was fitted for each CG:

*Y*_*ijkl *_= μ + *d*_*i *_+ *t*_*j *_+ *p*_*k *_+ *tp*_*jk *_+ ε_*ijkl*_

where *Y*_*ijkl *_is the transcript abundance for dye *i*, treatment *j*, probe set *k*, and replicate *l*; μ is the overall mean of the transcript abundance for that CG; *d *is the dye effect; *p *is the effect of probe set; and ε is the error. A treatment (*t*) in this case is a combination of line and sex, and there were a total of 16 treatments since we examined 2 sexes for each of 8 lines. The ANOVA modeling approach compares means among groups, and determines whether the means are significantly different given the observed level of variation. To test whether a particular effect was statistically significant, we used the FDR approach [80], which is common in genomic research [81-85] (an introduction can be found in [86]). Briefly, the false discovery rate controls the proportion of false positives in the total list of tests rejected. Thus, if 100 tests are rejected, and the FDR is set to 0.05, the expected number of false positives is 5. First, we tested the main effect of treatment (*t*_*j*_). That is, we tested whether the means were different among any of the 16 line/sex combinations (treatments). If this test was significant at FDR = 0.05, we declared this gene significant and investigated further whether the differences were due to sex, line, or interaction between sex and line effects at a very strict FDR of 0.05/3. To determine whether the relative amounts of alternative transcripts differed among sexes or lines, we tested the interaction between probe set and treatment (*tp*_*jk*_) and, if it was significant at FDR = 0.05, we further examined whether this was due to interaction between probe and sex or probe and line effects. For cases where the main effect of probe set (*p*_*k*_) was significant, we compared the effect of 'global' probes to each 'local' probe. The multiple transcript model was also fitted for gene families.

Significant probe-by-sex or probe-by-line interactions might arise not only as a consequence of genetic variation in alternative transcript production, but also as an artifact of intrinsic differences between probes. In order to estimate the rate of such artifacts, we used the model above to examine sets of probes that were expected to hybridize to the same transcript or group of transcripts (that is, the same unique region or the same common region). For such sets of probes, their relative intensities should, in principle, be identical in all treatments, and thus no significant probe by treatment interactions should be observed. By measuring the actual proportion of the control probe sets for which probe by treatment interaction is significant, we can estimate the rate of putative false positives. However, it should be noted that the expected hybridization targets of the probe sets are defined based on the current annotation, and it is possible that some of the probes are in fact hybridizing to different transcripts or sets of transcripts. Thus, this approach will probably over-estimate the number of false positives.

For genes that had a single informative transcript, the following ANOVA model for single transcripts was fitted for each transcript individually:

*Y*_*ijl *_= μ + *d*_*i *_+ *t*_*j *_+ ε_*ijl*_

Where Y_ijl _is the transcript abundance for dye *i*, treatment *j*, and replicate *l*; μ is the overall mean of the transcript abundance for that transcript; *d *is the dye effect; and ε is the error. As above, a treatment (*t*) in this case is a combination of line and sex, and there were a total of 16 treatments since we examined two sexes for each of 8 lines. [87-92]. Significance testing was performed as described above. All analyses were performed using SAS software version 9.1 (SAS Institute, Cary, NC, USA).

### Quantitative PCR analysis for data validation

Total RNA was isolated from whole virgin adults of the *Oregon-R *line as described above. For each sex, we used three biological replicates of four individuals each. To correct for differences in transcript abundance between sexes, samples were equalized by evaporation and resuspension in DEPC-treated water (DEPC: Diethyl pyrocarbonate). DNase I digestion (NEB, Ipswich, MA, USA) was carried out for 30 minutes at 37°C. Reverse transcription was performed on 5 μg of total RNA using oligo(dT)_16_, as described by the manufacturer (Applied Biosystems, Foster City, CA, USA). qPCR was performed on 100 ng of cDNA product in a total volume of 25 μl using TaqMan PCR Mix (Applied Biosystems). Primers for qPCR were designed to amplify either constitutive or alternative exon junctions of specific transcripts listed in Additional file 6. PCR amplification was detected using SYBR^® ^Green I dye chemistry and ABI Prism 7900 Real Time PCR system (Applied Biosystems). CTs were determined using the AB7900 system SDS software and defined as the fluorescence intensity significantly above background during the exponential phase of amplification for all reactions. For each gene, CT values were analyzed using the ANOVA model:

*Y*_*ijk *_= μ + *s*_*i *_+ *p*_*j *_+ *sp*_*ij *_+ ε_*ijk*_

where *Y*_*ij *_is cycle count for the *i*^*th *^sex and *j*^*th *^transcript for replicate *k*; μ is the overall mean for that gene and ε is the random error. Specifically, we tested whether the sex by transcript interaction effect was significant at a nominal level of 0.05.

All programs developed during this work [93] as well as the oligonucleotide sequences [94] are freely available.

## Additional data

The following additional data are available with the online version of this paper. Additional data file 1 includes the microarray results for several sets of genes for which we had *a priori *expectations of sex-biased expression. Additional data file 2 includes the processed microarray data used for analysis, as well as annotation from FlyBase from our AAP program. Actual_set_id is the unique identifier for each probe that hybridizes to the same set of transcripts, and actuals_cluster_id is a unique identifier that groups probes based upon CG number. Probeuid is the unique identifier for that probe sequence. Additional data file 3 provides results of the analysis, as well as annotations from FlyBase. The *P *values obtained from the ANOVA are given with the notation p<*effect*>. The CG number is given in actuals and the model used for analysis (Single transcript/multiple transcript) is given in the final column. Additional data file 4 gives the results of analysis based upon the probe level, as well as annotations from FlyBase. Additional data file 5 provides the probe-by-probe analysis of alternatively spliced genes analyzed using ANOVA model for multiple transcripts. The columns are, in order: probe ID; gene name; whether hybridization signal detected by that probe is greater in males or females; log-transformed female/male expression ratio for each probe; probe set ID; class of probe (global or local); *P *value for the sex by probe set interaction; and the list of transcripts targeted by each probe. See text for further details. Additional data file 6 includes the qPCR validation of sex-specific splicing. We give the probe sequences used, all qPCR results as well as the original array results to facilitate comparison. The *P *values of the likelihood ratio test (LRT) for a significant probe-sex interaction are also given. Note that for genes where only one transcript was tested, the test of the interaction between transcript and sex is not applicable (NA).

## Supplementary Material

Additional data file 1 Microarray results for several sets of genes for which we had *a priori *expectations of sex-biased expression Click here for file

Additional data file 2 Actual_set_id is the unique identifier for each probe that hybridizes to the same set of transcripts, and actuals_cluster_id is a unique identifier that groups probes based upon CG number. Probeuid is the unique identifier for that probe sequence. Click here for file

Additional data file 3 The *P *values obtained from the ANOVA are given with the notation p<*effect*>. The CG number is given in actuals and the model used for analysis (Single transcript/multiple transcript) is given in the final column. Click here for file

Additional data file 4 Results of analysis based upon the probe level, as well as annotations from FlyBase Click here for file

Additional data file 5 The columns are, in order: probe ID; gene name; whether hybridization signal detected by that probe is greater in males or females; log-transformed female/male expression ratio for each probe; probe set ID; class of probe (global or local); *P *value for the sex by probe set interaction; and the list of transcripts targeted by each probe. See text for further details. Click here for file

Additional data file 6 We give the probe sequences used, all qPCR results as well as the original array results to facilitate comparison. The *P *values of the Likelihood Ratio Test for a significant probe-sex interaction are also given. Note that for genes where only one transcript was tested, the test of the interaction between transcript and sex is not applicable (NA). Click here for file

## Figures and Tables

**Table 1:** Results from ANOVA models for single and multiple transcripts for the set of 10,933 detected genes

	Multiple transcript model			Single transcript model				Total
	ALTS	GF	Total	S	ALTS	GF	Total	

Number of genes	828	91	919	8,292	1,651	71	10,014	10,933
Number significant for treatment	653	79	732	4,613	818	39	5,470	6,202
Number significant for line	202	27	229	297	48	4	349	578
Number significant for sex	544	67	611	4,393	792	36	5,221	5,832
Female biased	249	31	280	2,352	552	16	2,920	3,200

Singletons (S) with multiple probes to the same transcript are included in the singleton category. Alternative splice variants (ALTS) and gene families (GF) were analyzed as multiple transcripts only when more than one probe was detected and otherwise these were analyzed as single transcripts.

**Table 2:** Probe targets and effects of sex and sex by probe interaction for several components of the sex determination pathway

Genes	Probes	Transcripts			Ratio (female/male)*	Sex effect^†^	Sex-by-probe
		Non-sex specific	Male	Female			

*tra *(CG16724)	9,174	-	-	-RA	1.10	<0.0001	NA
*tra2 *(CG10128)	4,734	-RA -RB -RD -RF -RG	-RC -RE	-	1.19	<0.0001	0.43
	11,136	-RA -RB -RF -RG	-RC	-	1.13	<0.0001	
	15,655	-RA -RB -RD -RF -RG	-RC -RE	-	1.11	<0.0001	
	19,044	-RA -RB -RD -RF -RG	-RC -RE	-	1.13	<0.0001	
*Sxl *(CG33070)	782	-RA -RC -RE -RG	-RB -RF -RK	-RD -RH -RJ	1.13	<0.0001	< 0.0001
	824	-RG	-RB		0.98	0.55	
	3,315	-RC		-RJ	1.12	<0.0001	
	4,972	-RC		-RJ	1.03	<0.0001	
	20,747	-RA -RC -RE -RG	-RB -RF -RK	-RD -RH -RJ	1.14	<0.0001	
*dsx *(CG11094)	6,162	-	-	-RB	1.25	<0.0001	0.0006
	12,495	-	-RA	-RB	1.06	0.22	
	12,690	-	-RA	-	0.95	0.20	
	13,818	-	-RA	-RB	0.99	0.93	
*her *(CG4694)	4,988	-RA	-	-	1.12	<0.0001	NA
*fru *(CG14307)	1,388	-RI -RJ -RL -RM	-RE	-RC	0.98	0.77	0.01
	2,271	-RI -RJ -RL -RM	-RE	-RC	0.94	0.05	
	9,294	-RB -RF	-	-	0.96	0.16	
	11,005	-RI -RJ -RL -RM	-RE	-RC	0.86	<0.0001	
	15,704	-	-RB -RE -RG	-RC -RF -RH	1.02	0.15	
	17,247	-	-RB	-RF	0.96	0.003	
	17,741	-RA -RD -RI -RJ -RK -RL -RM	-RB -RE -RG	-RC -RF -RH	1.03	0.071	
*Yp1 *(CG2985)	13,974	-	-	-RA	1.37	<0.0001	NA
*Yp2 *(CG2979)	13,101	-	-	-RA	1.68	<0.0001	NA
*Yp3 *(CG11129)	2,812	-	-	-RA	1.57	<0.0001	NA

A sex by probe interaction occurs when the relative amount of the two (or more) probes differs between males and females. Thus, if only one probe was present, then the sex by probe effect is not applicable (NA). *Ratios were estimated for each probe from the natural log of the background corrected signal. ^†^Individual probes were tested for difference between the males and females (sex effect) according to the single transcript model.

**Table 3:** Genes with probes targeting two or more non-identical sets of transcripts expressed

Classification of probes	Number of genes total (alternatively transcribed)	Significant total (alternatively transcribed)
Local probes only	89 (55)	25 (19)
Global + 1 local probe	608 (571)	103 (108)
Global + 2 local probes	151 (135)	36 (33)
Global + 3 local probes	46 (43)	14 (14)
Global + 4 local probes	12 (12)	5 (5)
Global + 5 local probes	8 (8)	2 (2)
Global + 6 local probes	3 (3)	0 (0)
Global + 7 local probes	1 (0)	0 (0)
Total	919 (828)	186 (182)

The genes with probes targeting two or more non-identical sets of transcripts expressed are divided into groups depending on the types of probes detected. The distribution of the type of probes detected for each gene are given as well as the number of these genes that show a significant interaction between the probe and the effect of either line or sex.

**Table 4:** Reliability of arrays (weighted kappa values [79]) based upon 20,265 probe spots

Comparison	Min	Q1	Median	Q3	Max
Overall (probes representing genes)	0.77	0.84	0.86	0.88	0.92
Alternative transcripts	0.78	0.84	0.87	0.88	0.92
Gene families	0.78	0.84	0.87	0.88	0.92
Pseudo clusters	0.70	0.81	0.84	0.87	0.96
Singletons	0.76	0.83	0.86	0.87	0.91
GC percentage	0.70	0.83	0.86	0.88	0.93
Tm	0.70	0.83	0.86	0.88	0.93
1 expected probe per cluster	0.77	0.84	0.86	0.88	0.92
2 to 5 expected probes per cluster	066	0.84	0.87	0.89	0.93
Suboptimal probes	0.74	0.80	0.83	0.85	0.90
1 transcript per probe	0.77	0.83	0.86	0.88	0.92
2 to 5 transcripts per probe	0.77	0.85	0.87	0.89	0.95

**Table 5:** Sex-biased expression of splicing regulators

CG#	Symbol	FlyBase ID	Ratio (F/M)	*P *(sex)	FDR
CG10279	Rm62	FBgn0003261	1.22	1.99 × 10^-29^	<0.05
CG10851	*B52*	FBgn0004587	1.22	1.36 × 10^-25^	<0.05
CG5442	*SC35*	FBgn0040286	1.20	6.67 × 10^-24^	<0.05
CG10445	*CG10445*	FBgn0037531	1.55	1.12 × 10^-23^	<0.05
CG16901	*sqd*	FBgn0003498	1.09	6.95 × 10^-22^	<0.05
CG8144	*ps*	FBgn0026188	0.87	6.48 × 10^-21^	<0.05
CG9696	*dom*	FBgn0020306	1.17	1.44 × 10^-19^	<0.05
CG5728	*CG5728*	FBgn0039182	0.80	3.09 × 10^-18^	<0.05
CG7437	*mub*	FBgn0014362	1.21	1.14 × 10^-17^	<0.05
CG7185	*CG7185*	FBgn0035872	1.33	3.34 × 10^-17^	<0.05
CG11360	*CG11360*	FBgn0039920	1.27	4.42 × 10^-14^	<0.05
CG12759	*Dbp45A*	FBgn0010220	1.23	4.66 × 10^-14^	<0.05
CG16941	*CG16941*	FBgn0038464	1.21	6.94 × 10^-14^	<0.05
CG4528	*snf*	FBgn0003449	1.21	2.15 × 10^-13^	<0.05
CG6841	*CG6841*	FBgn0036828	1.28	2.4 × 10^-13^	<0.05
CG4602	*Srp54*	FBgn0024285	1.23	7.93 × 10^-13^	<0.05
CG6197	*CG6197*	FBgn0033859	1.26	1.31 × 10^-12^	<0.05
CG12924	*CG12924*	FBgn0033450	1.25	1.57 × 10^-12^	<0.05
CG6999	*CG6999*	FBgn0030085	0.79	2.11 × 10^-12^	<0.05
CG3193	*crn*	FBgn0000377	1.23	2.14 × 10^-11^	<0.05
CG13425	*bl*	FBgn0015907	1.06	5.31 × 10^-11^	<0.05
CG9998	*U2af50*	FBgn0005411	1.17	5.94 × 10^-11^	<0.05
CG12749	*Hrb87F*	FBgn0004237	1.13	6.24 × 10^-11^	<0.05
CG3582	*U2af38*	FBgn0017457	1.22	1.15 × 10^-10^	<0.05
CG31762	*aret*	FBgn0000114	1.08	2.23 × 10^-10^	<0.05
CG5422	*Rox8*	FBgn0005649	1.23	1.63 × 10^-09^	<0.05
CG8019	*hay*	FBgn0001179	1.18	5.74 × 10^-09^	<0.05
CG5454	*CG5454*	FBgn0038667	1.14	1.38 × 10^-08^	<0.05
CG10418	*CG10418*	FBgn0036277	1.17	2.12 × 10^-08^	<0.05
CG8749	*snRNP70K*	FBgn0016978	1.16	5.44 × 10^-08^	<0.05
CG14641	*CG14641*	FBgn0037220	1.11	3.31 × 10^-07^	<0.05
CG2926	*CG2926*	FBgn0037344	1.10	3.57 × 10^-06^	<0.05
CG10210	*tst*	FBgn0039117	1.15	4.91 × 10^-06^	<0.05
CG9075	*eIF-4a*	FBgn0001942	1.06	2.79 × 10^-05^	<0.05
CG12085	*pUf68*	FBgn0028577	1.04	0.006741	<0.05
CG1646	*CG1646*	FBgn0039600	1.03	0.050962	<0.10
CG1658	*Doa*	FBgn0053553	0.99	0.306628	>0.20

The CG number, symbol and Flybase ID are given. The ratio (female/male (F/M)) is a ratio of log transformed signal values (natural log). *P *(sex) is the *P *value for the test of the null hypothesis that the males and females have the same amount of transcript. FDR gives the level at which that *P *value would be significant according to the Benjamini and Hochberg 1995 criteria [80].

**Table 6:** Microarray design

	Total number of probes	Total number of genes	Probes detected	Genes detected
Singletons 1 probe	12,912*	12,912	8,265	8,265
Singletons >1 probe	82	37	45	27
Gene families	566	177	436	162
Alternative transcripts	7,207	2,768	5,128	2,479
Negative controls	503	5	-	-
Agilent controls	1304	-	-	-
Total	22,574	15,894	13,874	10,933

The total number of probes and genes in each of the main categories, as well as the number of probes and genes detected in our experiment, are shown. *One singleton probe (of the same sequence) was printed in two duplicate spots.
